# Evaluation of Primary and Secondary Stability of Endosseous Dental Implants With and Without the Use of Platelet-Rich Fibrin: A Clinical Study

**DOI:** 10.7759/cureus.62918

**Published:** 2024-06-22

**Authors:** M. Tamilarasan, R. Nivetha, C.S. Prabhahar, M. Umayal, R. Arun Jaikumar, N. Madhulika Naidu

**Affiliations:** 1 Department of Periodontics, Best Dental Science College, Madurai, IND; 2 Department of Prosthodontics, Best Dental Science College, Madurai, IND; 3 Department of Oral Medicine and Radiology, Best Dental Science College, Madurai, IND

**Keywords:** resonance frequency analysis, implant stability, osseointegration, prf, implants

## Abstract

Background: This clinical study investigates platelet-rich fibrin's (PRF) impact on dental implant stability, addressing global oral health challenges and limitations of traditional methods. Emphasizing osseointegration's pivotal role, the study explores PRF's potential in enhancing implant stability, assessing it through resonance frequency analysis (RFA) and implant stability quotient (ISQ). The hypothesis suggests PRF may improve both primary and secondary stability, aiming to uncover clinical benefits in dental implant procedures

Materials and methods: The study involved 24 subjects from the Department of Periodontics outpatient clinics with a meticulously designed methodology. This included a pre-surgical protocol with oral prophylaxis, impressions, and cone-beam computed tomography (CBCT) analysis. PRF preparation utilized a minimally invasive venipuncture technique. Implant placement followed a two-stage surgical protocol, assessing primary stability with MEGA ISQ (Ostell). Post-surgery, patients received instructions and underwent recall for secondary stability after three months. Clinical parameters such as plaque index (PI), gingival index (GI), implant probing pocket depth (IPPD), sulcus bleeding index (SBI), and implant stability (IS) were systematically recorded. Robust statistical analyses, using IBM SPSS Statistics for Windows v20.0 (IBM Corp., Armonk, USA) software, incorporated Mann-Whitney U and Wilcoxon signed-rank tests for group and within-time point comparisons, with a significance level of p<0.05. This comprehensive study yields nuanced insights into the impact of PRF and implant procedures on key clinical parameters, contributing significantly to the field.

Results: This study compared dental implants with and without PRF in 24 patients. Both groups showed significant improvements in the PI, GI, and SBI. The PRF group exhibited higher IS in the third and sixth months, while IPPD was lower in the PRF group in the sixth month.

Conclusion: The findings of the study highlight a positive impact on implant stability contributing to better implant outcomes.

## Introduction

Global oral diseases impact public health, with a shift from caries to periodontal issues causing adult tooth loss [1,2] prompting a preference for implantology due to its effectiveness in addressing functional and aesthetic challenges [3]. Osseointegration, as defined by the American Academy of Implant Dentistry (AAID), involves direct implant-bone contact for load distribution. The Branemark system aligns with natural bone healing, boasting a 95% success rate for approximately 450,000 dental implants placed annually [4,5].

Platelet-rich products like platelet-rich growth factor (PRGF), platelet-rich plasma (PRP), and platelet-rich fibrin (PRF) are crucial in oral surgery for tissue regeneration. Recent studies show their use on titanium implants enhances bone-to-implant contact and osseointegration [6,7]. PRF, a second-gen platelet concentrate, introduced in 2000, is a significant advancement. Its simple protocol creates a fibrin scaffold, releasing growth factors for effective peri-implant bone healing [8,9].

Implant stability, indicating osseointegration, involves primary and secondary stability [10,11]. Primary stability, influenced by bone density and implant factors, is achieved during placement. Secondary stability depends on bone remodeling and is affected by surface characteristics [12,13]. Resonance frequency analysis (RFA), a non-invasive method introduced in 1996, measures stability with an implant stability quotient (ISQ), where >65 is successful and <50 suggests potential failure [10,14-16]. This study hypothesizes that PRF has the potential to enhance implant stability.

## Materials and methods

Data collection

This prospective clinical trial involved 24 participants recruited from patients attending outpatient clinics at the Department of Periodontics, Best Dental Science College and Hospital, Madurai, Tamil Nadu, India. The sample size was calculated using the formula:

n=[(Z_α/2_​+Z_β​​_)/d]^2^⋅(s_^1^_^2^​+s_2_^2^​),

where n = sample size per group; 𝑍_𝛼/2_​ = Z-value for the desired level of confidence (e.g., 1.96 for 95% confidence); 𝑍_𝛽_​ = Z-value for the desired power (e.g., 0.84 for 80% power); d = the minimum clinically significant difference between the two means; s_1​_ and s_2 _= standard deviations of the two groups.

Inclusion criteria encompassed individuals aged between 18-60 years, healed edentulous alveolar ridge should have received at least one dental implant, with suitable height and width to avoid dehiscence and fenestration, and the presence of opposing teeth. Exclusion criteria involved sites with acute infection, insufficient bone volume, patients with parafunctional habits, current smokers, pregnant or lactating women, and medically compromised patients.

Study design

Patients meeting inclusion/exclusion criteria underwent pre-surgery oral preparation, including prophylaxis and impressions. A surgical template ensured precise osteotomy, guided by preoperative cone-beam computed tomography (CBCT) for optimal implant size and placement.

Before stage I surgery for implant placement, a comprehensive hemogram, including hemoglobin mg%, bleeding time, clotting time, differential count, total count, and international normalized ratio (INR) count, was conducted. The pre-surgical protocol involved individualized study models, occlusal analysis done by using casts and articulating papers to check for any discrepancies, and the creation of surgical stents. Patients received oral prophylaxis one week prior, coupled with a seven-day regimen of chlorhexidine gluconate 0.2% mouthwash due to its superior antibacterial efficacy. Clear instructions were provided to ensure effective oral hygiene leading up to the surgery.

PRF Preparation

PRF preparation began with a minimally invasive 21-gauge needle venipuncture, targeting the median cubital vein. Collected blood in 10 ml tubes was immediately centrifuged at 3000 rpm for 12 minutes. The resulting fibrin clot, approximately 5 ml, was extracted, cleansed of red blood cell remnants, and gently compressed in a PRF Box (GDC Platelet Rich Fibrin Box, India) for insertion into the prepared implant site. 

Implant Selection and Placement

**Figure 1:**
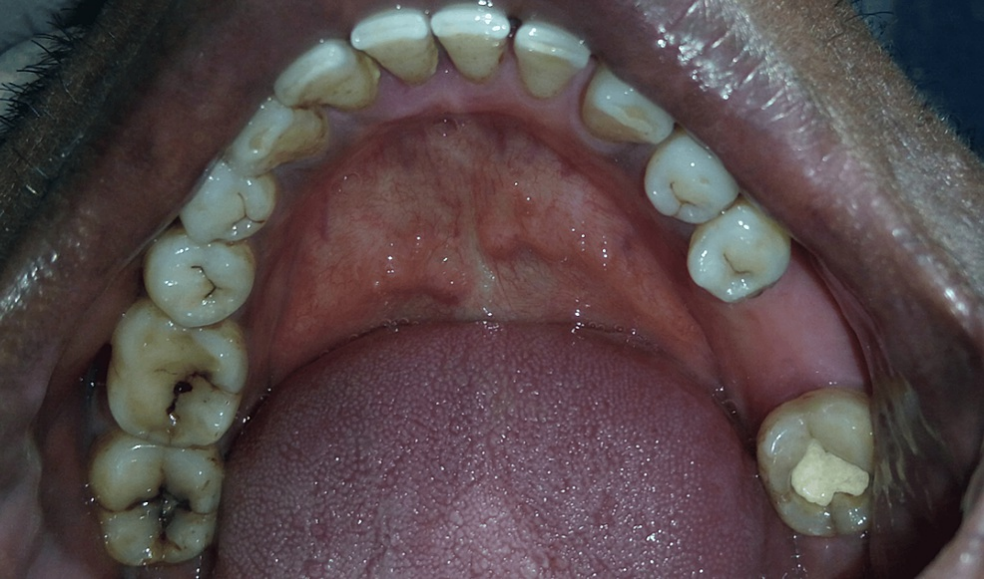
Preoperative view of the edentulous area

**Figure 2:**
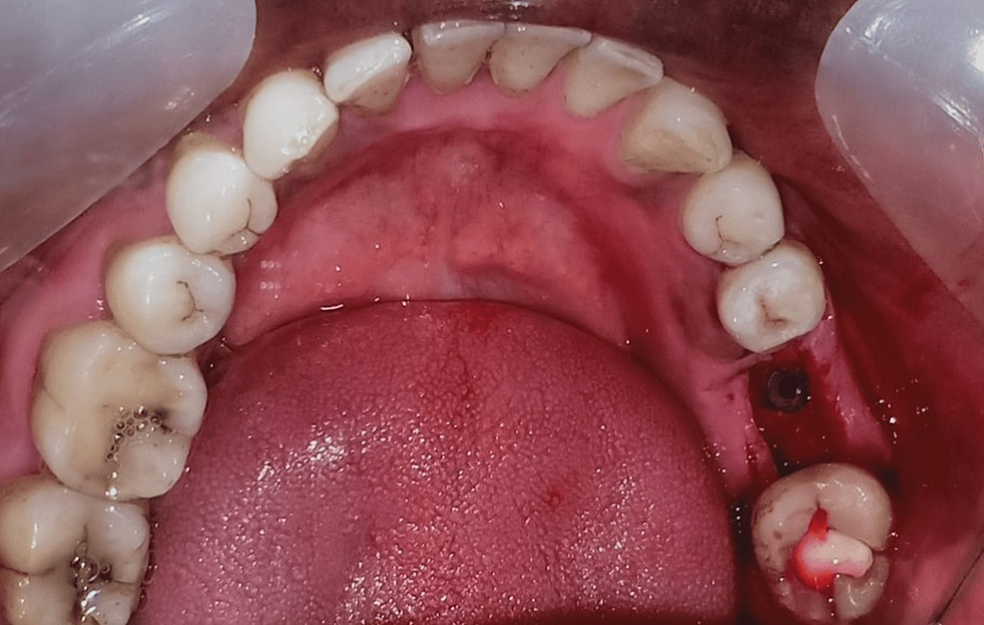
Implant placement in the patient

**Figure 3:**
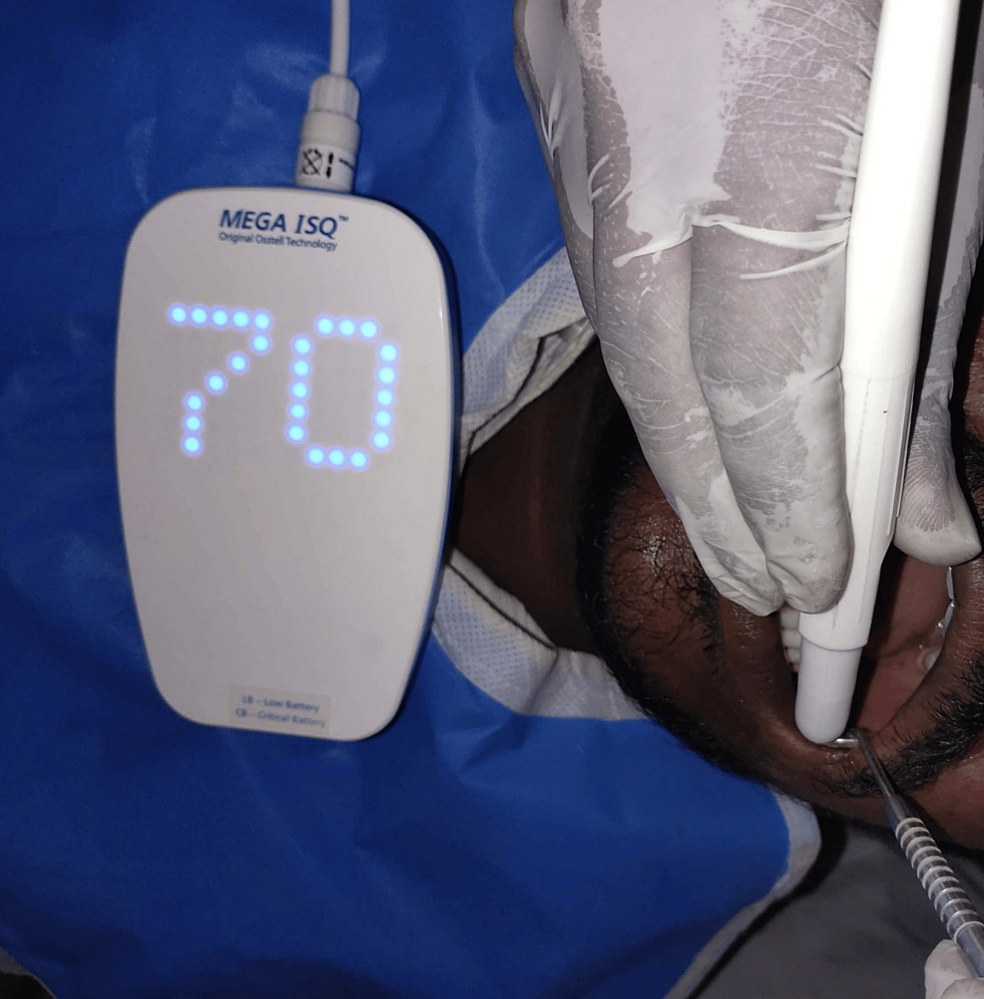
Primary stability checked for the implant

In the initial surgery stage, local anesthesia was administered, and implant sites were prepared using the NORIS® mini-surgical implant kit (NM-X2213; Noris Medical Ltd., Nesher, Israel) with mucoperiosteal flap reflection. The test group received a PRF clot during implant placement for enhanced stability while the control group did not. The primary stability for the implant was measured by MEGA ISQ (Osstell), with resonance ranging from 70 to 75 ISQ units. Using a frequency analysis device, two readings of the RFA values were recorded in bucco-lingual and mesio-distal directions (Figures 1-3).

**Figure 4:**
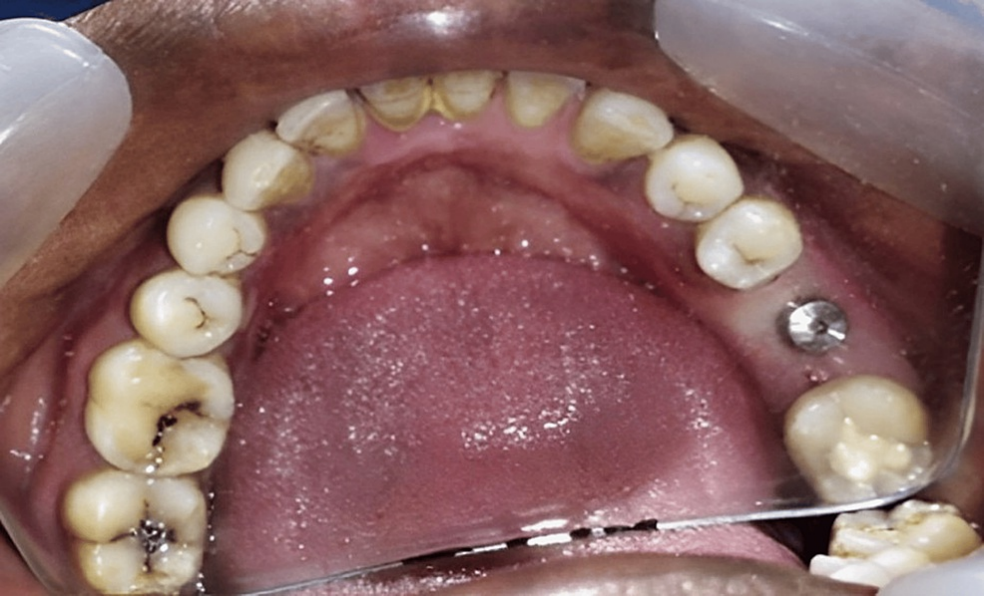
Implant placed in the patient

**Figure 5:**
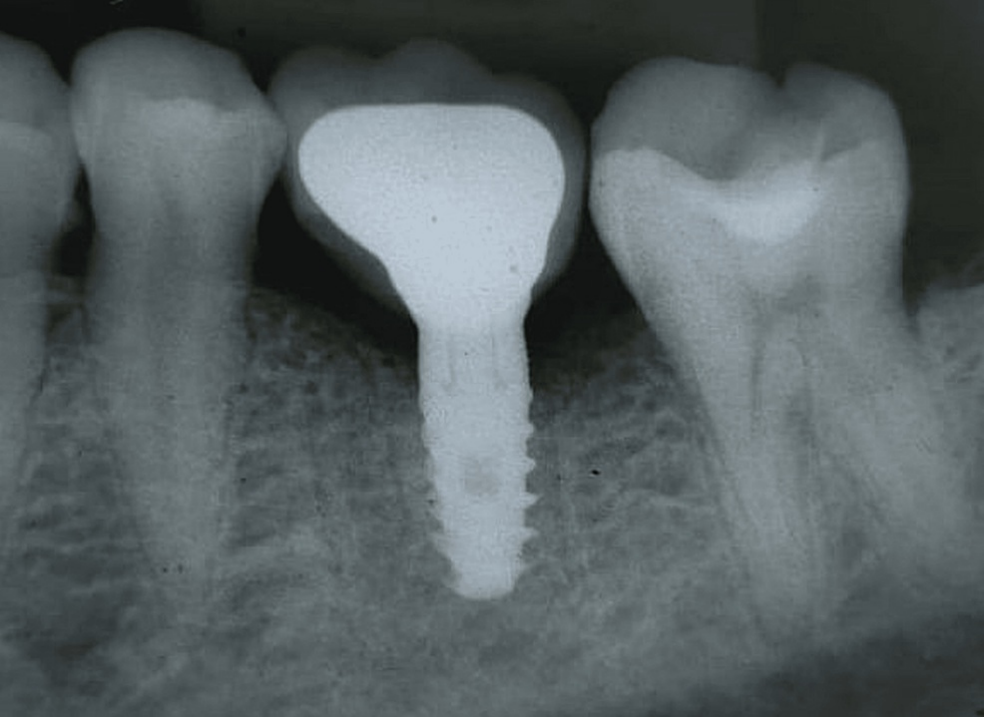
Radiograph of the implant placed after prosthesis

Post-surgery, patients followed prescribed medications, oral hygiene, and dietary instructions. In the second stage, conducted after three months, soft tissue was excised with gingival former placement, secondary stability was measured, and a screw-retained ceramic prosthesis was delivered, with proper adjustments made after one week (Figures 4-5).

Clinical Parameters Evaluation

All the subjects, including the control and test group, were evaluated for clinical parameters like plaque index (PI), gingival index (GI), implant probing pocket depth (IPPD) by using an Implant Probe (Hu-Friedy, USA), sulcus bleeding index (SBI), and implant stability (IS) at baseline, three months, and six months, respectively.

Data Analysis

Data was compiled in Microsoft Excel (Microsoft Corp., Redmond, USA) and analyzed using IBM SPSS Statistics for Windows v20.0 (IBM Corp., Armonk, USA). Mann-Whitney U tested intergroup variables (PI, GI, SBI, IPPD, IS). Wilcoxon signed-rank assessed intragroup changes over time. Significance was set at p<0.05. Normality was confirmed with Kolmogorov-Smirnov and Shapiro-Wilk tests; non-normally distributed data was analyzed using the Kruskal-Wallis test.

Ethical considerations

This study was approved by the Scientific and Ethical Committee review board of Best Dental Science College and Hospital, Madurai, Tamil Nadu, India. Patient confidentiality was maintained throughout the study, and all data were anonymized and securely stored.

## Results

**Figure 6:**
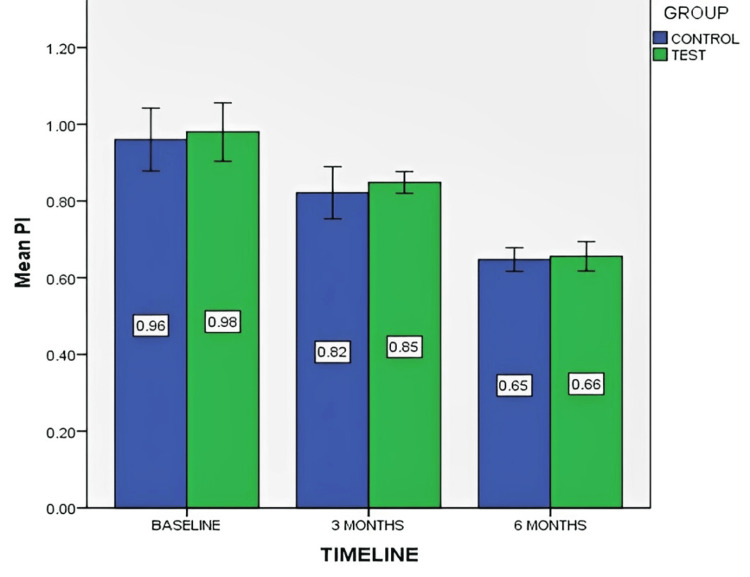
Mean plaque index (PI) score at baseline, three months, and six months for both control and test groups The data is represented as the mean value.

Figure 6 depicts the mean PI score at baseline, three months, and six months for both control and test groups.

The study compared PI scores between control (without PRF) and test (with PRF) groups over six months. While the Mann-Whitney U test showed no significant between-group differences (p-values: 0.52, 0.88, 0.68), within-group Wilcoxon signed-rank tests revealed statistically significant reductions (p-values <0.05) in PI at all time points for both groups, emphasizing the effectiveness of the intervention.

**Figure 7:**
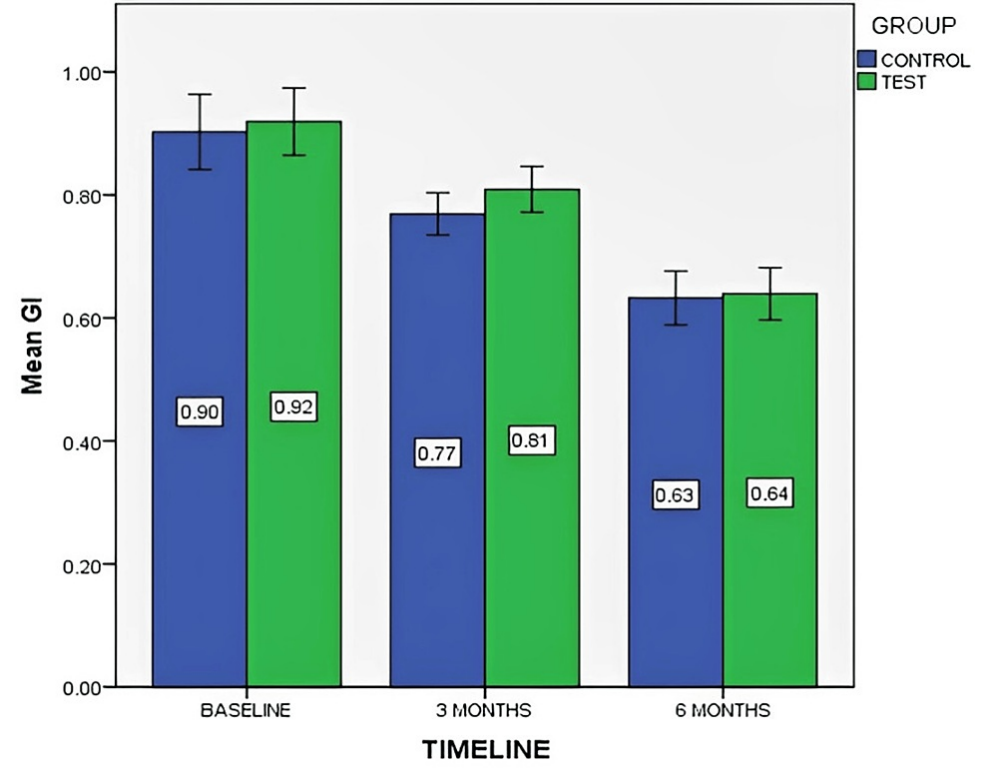
Mean gingival index (GI) score at baseline, three months, and six months for both control and test groups The data is represented as the mean value.

Figure 7 depicts the mean GI score at baseline, three months, and six months for both control and test groups.

It showed reductions in mean GI scores at three and six months for both the control group (0.90 to 0.76 and 0.63) and the test group (0.91 to 0.80 and 0.63). The Wilcoxon signed-rank test showed significant improvements within both groups: control (p-values: 0.008, 0.003, 0.002) and test (p-values: 0.011, 0.002, 0.003) at various intervals. However, the Mann-Whitney U test demonstrated no significant differences between control and test groups at baseline, three months, and six months (p-values: 0.60, 0.10, and 0.81). Overall, while both groups internally improved, there was no statistically significant advantage of PRF in the between-group comparison.

**Figure 8:**
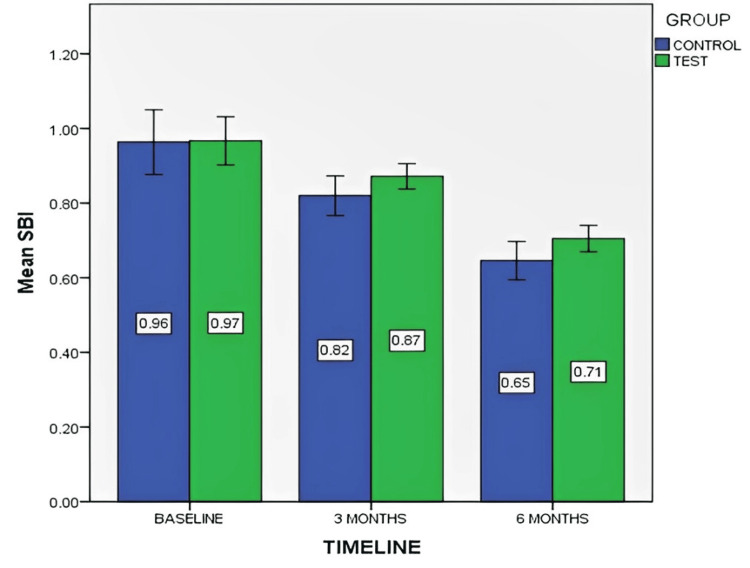
Mean sulcus bleeding index (SBI) score at baseline, third month, and sixth month for both control and test groups The data is represented as the mean value.

Figure 8 depicts the mean SBI score at baseline, three months, and six months for both control and test groups.

The study compared the SBI between control (without PRF) and test (with PRF) groups over three time points. A significant reduction in SBI was observed in both groups in the sixth month. The between-group Mann-Whitney U test showed a significant difference at six months (p=0.045). Within-group Wilcoxon signed-rank test revealed significant improvements in SBI at all time points for both control and test groups (p<0.05).

**Figure 9:**
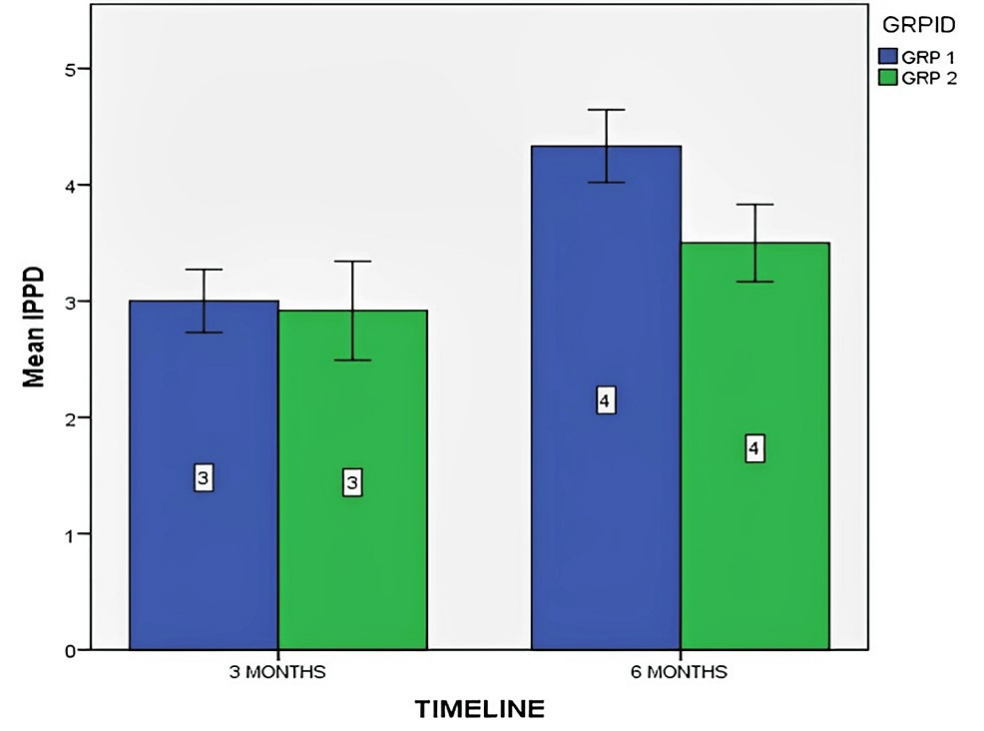
Mean implant probing pocket depth (IPPD) at three and six months for both control and test groups The data is represented as the mean value.

Figure 9 depicts the mean IPPD at three and six months for both control and test groups.

IPPD was compared between the control (without PRF) and test (with PRF) groups over two time intervals. A significant increase in depth was noted in both groups in the sixth month, with the Mann-Whitney U test showing statistical significance (p=0.005). The within-group Wilcoxon signed-rank test indicated significant changes at both time points for both control and test groups (p<0.05).

**Figure 10:**
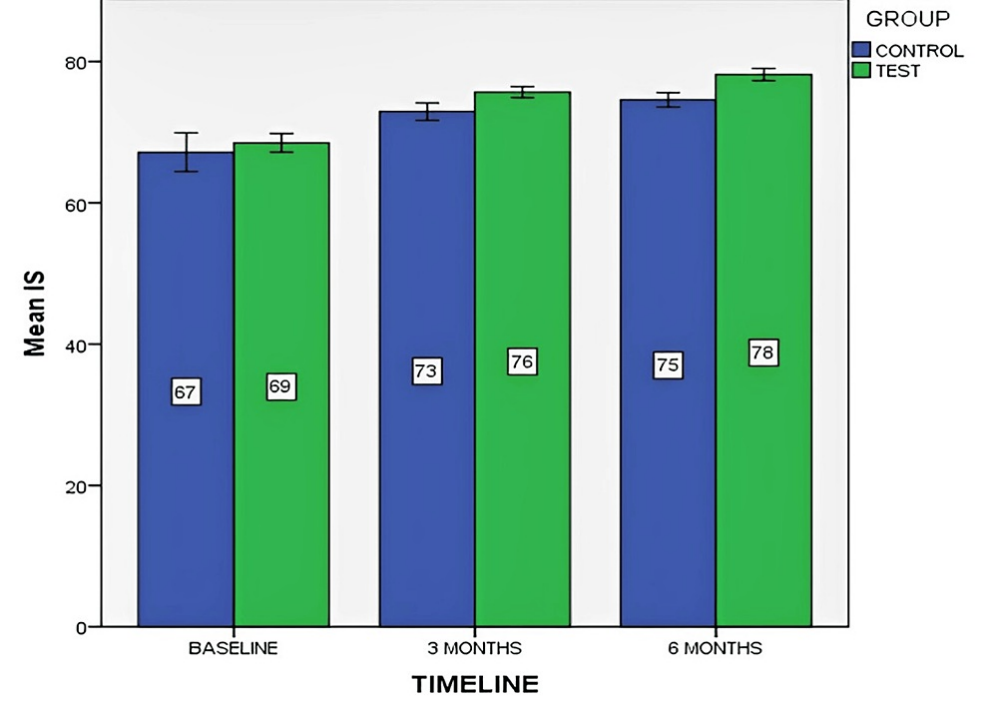
Mean implant stability (IS) at baseline, three months, and six months for both control and test groups The data is represented as the mean value.

Figure 10 depicts the mean IS at baseline, three months, and six months for both control and test groups.

IS was compared between the control (without PRF) and test (with PRF) groups at various time intervals. Significant increases in IS were observed in the third and sixth months for both groups. Mann-Whitney U test indicated significant between-group differences at both time points (p=0.000). The within-group Wilcoxon signed-rank test showed significant improvements at all time points for both control and test groups (p<0.05).

## Discussion

To enhance dental implant success, strategies like modifying surface topography and using patient-derived platelet-based preparations, such as PRF, promote osseointegration and long-term efficacy by activating the vascular system and releasing growth factors for comprehensive tissue healing [17,18]. PRF, a second-gen autologous platelet concentrate, forms a fibrin mesh with leukocytes, cytokines, and stem cells, playing a pivotal role in angiogenesis and supporting both soft and hard tissue healing [9,19].

Implant stability, indicative of osseointegration, is traditionally assessed through methods like histologic analysis and various clinical tests. RFA is a non-invasive and reliable method for measuring implant stability. The study on 24 patients, comparing dental implants with and without PRF using RFA, evaluated parameters such as GI, PI, SBI, and IS at baseline, three months, and six months. Oral hygiene status remained satisfactory, with statistically similar values between groups, emphasizing the importance of good oral hygiene in maintaining implant stability.

DeAngelo et al. (2007) stressed probing depth for soft tissue maturity [20], while Dierens et al. (2012) and Winitsky et al. (2018) found healthy peri-implant mucosa often exceeds 4 mm (60-63%) and 6 mm (15-23%) [21,22]. In our study, probing depth stayed below 4.5 mm, and significant group differences emerged in the sixth month, underscoring its role in assessing soft tissue response. Mean values within groups in the third and sixth months showed statistically significant changes in implant probing depth.

Implant stability, measured by RFA, revealed a mean baseline stability of 67.17 ± 4.32 ISQ for the control group and 68.50 ± 2.06 ISQ for the test group (with PRF), aligning with the findings of Ostman et al. (2006) with a mean ISQ value of 67.4 (SD 8.6) for 905 Branemark dental implants [23]. Sennerby and Meredith (2002) defined ISQ ≥ 70 as high stability, predicting a small drop in stability levels if the initial ISQ is high [22]. In the third month, mean implant stability was 72.90 ± 1.92 ISQ for the control group and 75.67 ± 1.23 ISQ for the test group, aligning with findings by Zhou et al. (2009) [24]. In the sixth month, stability increased, with mean ISQ values of 74.58 ± 1.62 for the control group and 78.17 ± 1.33 for the test group, consistent with studies by Bornstein et al. (2009) [25], Tabrizi et al. (2017) [26], and Torkzaban et al. (2018) [27]. In contrast with the present study, other studies concluded that local application of PRF exhibited no statistically beneficial effect on implant stability. This was reported by Hussien et al. in 2017 [28].

The study's limitations include a relatively small sample size, potentially limiting the generalizability of findings. Additionally, the six-month follow-up period might not capture long-term outcomes adequately. Conducting the study at a single center may have introduced biases, and the lack of blinding could affect the assessment of clinical parameters. While the study assessed various clinical parameters, the absence of patient-reported outcomes and radiographic assessments limits the comprehensiveness of the findings. Addressing these limitations in future research could provide a more nuanced understanding of PRF's impact on dental implant procedures and outcomes.

## Conclusions

This study compared dental implant stability with and without PRF in 24 patients. While plaque and gingival indices remained consistent, PRF showed significant benefits in reducing sulcus bleeding at six months and improving implant stability at both three and six months. Thus, PRF application during implant surgery demonstrated statistical advantages for stability, emphasizing the need for extended clinical trials and histological studies for comprehensive validation.
